# Supplementary material for: Selective agonism of liver and gut FXR prevents cholestasis and intestinal atrophy in parenterally fed neonatal pigs
Source: J Lipid Res. 2025 Oct 1;66(11):100919. doi: 10.1016/j.jlr.2025.100919 (PMC12637226; doi:10.1016/j.jlr.2025.100919)
Supplement: Supplemental Material Table 1 [file mmc1.docx]

| **Supplemental Material Table 1.** Forward and reverse primer sequences used for qRT-PCR. | | |
| --- | --- | --- |
| Gene | Fwd | Rev |
| *NR1H4* | TTTGTGTCGTTTGCGGAGAG | GTTGCCCCCATTTTTACACTTG |
| *NR0B2* | GCCTACCTGAAAGGGACCAT | CAACGGGTGTCAAGCCTTTA |
| *FGF19* | CTGGGCCGCACGTGCACTAC | GGGCCCGTCTGAGTGGATGCG |
| *ABCB11* | TTTCATTCAGCGCCTGACCA | ACTCCAATGAGAGGGCTGAC |
| *ABCB1* | TATAACACCAGAGTGGGAG | GACAACCTTTTCACTTTCTG |
| *ABCC4* | CTTTGCCAATGCACTCCTTG | ACTCTTTGGATGCTGACGAC |
| *SLC51A* | TGTACAAGAACACTCGCTGC | GAACACACACACTATCGTGGG |
| *SLC10A2* | CTTTCGGAAACCTAAGGGACT | AAGAGCTTGCCCAGTGCAAAG |
| *ABCG5* | TCCAATGTGTGCCTTGAGTC | CCAGGATGACAAGAGTTGGG |
| *ABCG8* | GTCTGGCACCACCATCTACT | TCCACCCCATAGAAGTCAGC |
| *CYP7A1* | GAAAGAGAGACCACATCTCGG | GAATGGTGTTGGCTTGCGAT |
| *CYP3A29* | GTGGAGTGTTACATACGGGC | AGGTGATACTAGGTGGGGGT |
| *CYP4A21* | TTTTCCCGCTTGAGGAGTGC | ACTCGGTCTGTGTGTTGATGGA |
| *CYP8B1* | CCGGAAGAATATGTTGGAAT | AAGTCTAGTTTTCTCTTCGC |
| *CYP27A1* | ACTGAAGACCGCGATGAAAC | CAAAGGCGAATCAGGAAGGG |
| *CYP7B1* | AATACTTCCTCCCTTCTGCCC | GGGCAGAAGGGAGGAAGTATT |
| *FABP6* | CTCCTGCCTCATCCTTC | CATCGTAGTTCTTCTACTC |
| *SLC10A2* | ATAATGGGATGCTGTCCAGG | TAGATTAAGAGGCACAGCGG |
| *ACTB* | GGACCTGACCGACTACCTCA | GCGACGTAGCAGAGCTTCTC |
